# *Wohlfahrtiimonas chitiniclastica* Bacteremia Associated with Myiasis, United Kingdom

**DOI:** 10.3201/eid2106.140007

**Published:** 2015-06

**Authors:** Lisa Campisi, Nitin Mahobia, James J. Clayton

**Affiliations:** Royal Surrey County Hospital, Guildford, United Kingdom

**Keywords:** *Wohlfahrtiimonas chitiniclastica*, bacteria, bacteremia, *Lucilia sericata*, green bottle fly, myiasis, United Kingdom

**To the Editor:** We report the identification of *Wohlfahrtiimonas chitiniclastica* bacteria in a sample of blood obtained from a patient in Surrey, United Kingdom. We highlight the importance of recognizing unusual bacteria that are associated with the larvae of parasitic flies as a potential causative agent of severe infection in patients with myiasis in the United Kingdom and possibly worldwide.

The patient, an 82-year-old woman, was found collapsed in her garden with maggots covering her body and hair. Skin excoriations over her neck, face, and head showed superficial tissue breakdown in keeping with proteolytic enzyme secretions of maggots. The woman may have been lying outside for 72–96 hours. She had a history of recurrent falls, hypertension, chronic kidney disease, ischemic heart disease, hypercholesterolemia, and osteoarthritis.

Blood analysis showed a marked inflammatory response. The patient had a C-reactive protein level of 157 mg/L (reference <10 mg/L); leukocyte count of 15.56 × 10^9^ cells/L (reference 4.0–11.0 × 10^9^ cells/L); predominant neutrophilia; and evidence of rhabdomyolysis. She had persistent acute kidney injury; her creatinine level was 131 µmol/L (reference 49–90 µmol/L), and her urea level was 23.3 mmol/L (reference 2.5–7.8 mmol/L). Her serum lactate level was 2.5 mmol/L (reference 0.6–2.2 mmol/L), suggesting sepsis.

Intravenous antimicrobial drug therapy with cefuroxime (750 mg 3×/d), metronidazole (500 mg 3×/d), and clarithromycin (500 mg 2×/d) was continued for 7 days, followed by oral flucloxacillin (500 mg 4×/d). Topical chloramphenicol and fusidic acid were applied to ear canals. Superficial maggots were manually removed; however, larvae continued to emerge from the patient’s inflamed ear canals, requiring otoscopic removal and cleaning of her ears. Larvae were identified as the third instar of *Lucilia sericata*, the common green bottle fly. *W.* *chitiniclastica* bacteria, which were isolated from cultures of blood samples obtained on admission, were identified by using matrix-assisted laser desorption/ionization time-of-flight (MALDI-TOF) mass spectrometry (Bruker Daltonics, Billerica, MA, USA), a tool for rapid identification of uncommon microorganisms, and confirmed by using 16S rRNA sequencing. Two *W. chitiniclastica* reference isolates were in the MALDI-TOF database at time of testing; scores matched to our isolate were 2.264 and 2.200, indicating a match to species level. We could not isolate *W. chitiniclastica* from swab specimens of superficial lesions or ear swab specimens.

Blood culture samples grew a mixture of *Proteus mirabilis, Providencia rettgeri*, and S*taphylococcus aureus*. The patient made good clinical recovery and was later discharged to a local rehabilitation unit.

Previous case reports from Argentina and the south of France of bacteremia caused by *Wohlfahrtiimonas* spp*.* involved homeless persons with histories of alcohol abuse, 1 of whom was infested with insect larvae (*1**,**2*)*.* One of these patients died from sepsis. *W. chitiniclastica* is known to colonize at least 2 species of flies but is not reported in *Lucilia* sp. This bacterium has been isolated from larvae of the fly *Wohlfahrtia magnifica,* a serious parasite of livestock in eastern Europe, the Mediterranean, and Central Asia (*3*), but this fly is not usually seen in the United Kingdom. *W. chitiniclastica* has also been isolated in China from *Chrysomya megacephala* oriental latrine flies, a screwworm species common in tropical and subtropical regions that is a facultative cause of myiasis (*4**,**5*). A study from South Korea reported a new *Wohlfahrtiimonas* sp. isolated from the larval gut of *Hermetia illucens*, the black soldier fly, although this fly is not pathogenic (*6*)*.*

*L. sericata* is a blowfly that is common across much of the world. Although it usually feeds on dead or necrotic tissue, it can invade healthy tissue and is the cause of sheep blowfly strike (i.e., cutaneous myiasis) in otherwise healthy livestock. This organism has a role in forensic investigations and is used in health care settings for larval debridement of necrotic tissue from wounds and ulcers (*5*)*.* The woman in our study had myiasis (i.e., infestation) with some invasion of healthy tissue and tissue damage from enzymes secreted by the larvae.

In this case, use of MALDI-TOF mass spectrometry enabled rapid identification of a rare bacterial species (*7*) in a patient with myiasis; slower molecular methods were previously required for such diagnoses. Without local availability of this technology, considerably more time would have been required for the diagnosis. Previous lack of identification of this species may be due to the former shortage of *W. chitiniclastica* isolates in the MALDI-TOF database.

This case demonstrates association of *W. chitiniclastica* with myiasis, although the pathogenic role in this clinical situation is uncertain. It is difficult to ascribe the clinical symptoms solely to bacteremia caused by this organism because multiple organisms were isolated. The cultures might have been heavily contaminated, but this would still highlight an association between *L. sericata* and *W. chitiniclastica.* Although we did not test the extracted fly larvae for *W.* *chitiniclastica,* we believe it is likely that the bacteremia originated from the patient’s inner ear infestation. *L. sericata* may be a vector for this microorganism in the United Kingdom, and possibly worldwide, given this fly’s widespread habitat.
